# Australian Community and Health Professionals Perceptions of Equine-Assisted Psychotherapy

**DOI:** 10.1155/2021/2217761

**Published:** 2021-12-10

**Authors:** P. Stapleton, K. T. Grimmett

**Affiliations:** School of Psychology, Bond University, Gold Coast, Queensland 4229, Australia

## Abstract

Mental health conditions are increasingly prevalent in the Australian population, and despite the large evidence-based support for contemporary treatments, there are barriers which inhibit their efficacy. Thus, there is a perceived need for therapists to consider other therapeutic options which have potential to enhance treatment outcomes. There is increasing acceptance for complementary and alternative medicines (CAM) among general practitioners and clients/general community. Specifically, more than 70% of Australians utilize CAM. Equine-assisted psychotherapy (EAP) is an underutilized, culturally sensitive, complementary therapy, which has the potential to mitigate barriers of conventional therapy. The present study aimed to determine the level of knowledge about and general acceptance of EAP as a treatment for general psychopathology symptomology within community members and health professionals. The current sample included 144 community members and 55 health professionals, all with Australian citizenship. Data analysis comprised the independent *t*-test and two hierarchical multiple regressions. Results indicated that community members are significantly more accepting of EAP as a treatment compared to health professionals. Of the predictors tested, higher social support and openness within community members were significant predictors of accepting perceptions, and rural location was the only significant predictor for health professional's accepting perceptions of EAP. This is one of the first studies to investigate perceptions of EAP outside the EAP field and through comparison between community members and health professionals. The current study identifies the need for future research to further investigate perceptions of EAP among Australian health professionals.

## 1. Introduction

The World Health Organization [1] identifies mental health as ones' well-being status, characterized by the capacity to cope with typical life stressors, work productively, and contribute to their community, as well as the realization of ones' own potential. Poor mental health is associated with mental illness or mental disorders which are often apparent through some combination of atypical behaviours, emotions, thoughts, and relationships with others. Notably, mental health disorders encompass numerous problems, with varying symptoms. The Australia Bureau of Statistics 2014-2015 national report identified four million Australians (17.5%) as having a mental health condition [2]. Comparatively, the recent 2018 Australia Bureau of Statistics national report found one in five (20.1%), that is, 4.8 million Australians, was suffering a mental health condition. Moreover, the lifetime incidents of mental health conditions in the total Australian population are 45.5% [2].

The effects of mental illness often extend beyond the individual (sufferer) and impact profoundly on close family, friends, and colleagues. Hence, mental health issues permeate the wider community. The National Mental Health Commission [3] identified the cost of mental health issues in Australia as $4,000 AUD for each individual, per annum, which is a total of $60 billion AUD for the whole population per annum. A population's burden of disease is best understood as the number of years of healthy life lost by individuals as a result of premature death (fatal burden) or living with ill health (nonfatal burden). According to the Australian Institute of Health and Welfare [4], mental health and substance use disorders accounted for 12% of Australia's total burden of disease, ranking it the fourth highest (after cancer, cardiovascular diseases, and musculoskeletal conditions). Moreover, individuals in remote locations experience a heightened burden of disease, 1.4 times that of individuals in major cities. This is evident across multiple burden categories including mental health and substances disorders, as well as suicide and self-inflicted injuries.

Individuals who reside in remote and very remote locations typically have poorer access to health services compared to individuals in regional areas and major cities [5]. Furthermore, first nation Australians are a high-risk population for mental illness, and remote areas have higher per capital populations of people who are identified as Aboriginal and Torres Strait Islander [5]. In 2017-18, contacts for more than 9.5 million community mental healthcare services were provided to approximately 435,000 patients. Importantly, Aboriginal and Torres Strait Islander patients received the aforementioned services at around three times the rate of their nonindigenous counterparts (53.8 compared to 16.1 per 1,000 population, respectively). Despite this, there exists a substantial disparity of mental health burden on indigenous Australians (higher burden) compared with nonindigenous Australians (lower burden). Moreover, poor mental health is commonly associated with suicide. Men are at greater risk of suicide; however, they are the sector of the population which is least likely to seek help [6]. An estimated 72% of males with mental illness do not seek help. Combined, the statistics accentuate the need for more culturally sensitive therapies, particularly for first nation Australians, men, and individuals residing in rural locations.

### 1.1. Therapy

Treatment types for general psychopathology symptomology typically include psychological treatment (psychotherapy), medication, and complementary and alternative medicine (CAM; DSM-5, 2013; [7]). Best practice treatment is dependent upon the disorder classification and subsequently varies according to the individual, their history of illness, and the severity of the preexisting illness.

### 1.2. Conventional Therapy

Psychotherapy is the most utilized conventional treatments of general psychopathology symptomology [7]. Psychotherapy derives from established psychological principles and encompasses the purposeful and informed application of clinical techniques and interpersonal stances. It has the objective of assisting individuals in modifying their cognitive, behavioural, emotional, and/or other personal characteristics in directions deemed desirable by the individual [8]. There are a range of psychotherapies available, including cognitive behavioural therapy (CBT), exposure therapy (ET), and dialectical behaviour therapy (DBT). Although such variations exist within the conceptual underpinnings, psychotherapies typically consist of one-on-one sessions, between the client and practitioner, with some sessions conducted in groups, consisting of relationships or families. Traditional approaches are not always effective, for example, for clients to receive the full therapeutic benefits of CBT, they must undertake homework activities. Less than 50% of individuals complete such activities, and thus, these individuals may not experience therapeutic success [8]. Notably, the Diagnostic and Statistical Manual-Fifth edition (DSM-5) is the first edition to include cultural considerations. Therefore, previous diagnoses and subsequent treatments may not have yielded successful outcomes or at least reached their full therapeutic capacity due to the general insensitivity toward cultural influences/components (DSM-5, 2013).

Although the majority of psychotherapies are considered evidence-based for the treatment of particular mental disorders, some are criticized as pseudoscience [9]. Moreover, government subsidized programs such as Medicare only approve those therapies listed by the American Psychological Association (APA), thus excluding many alternative approaches, which may reduce a therapy's feasibility to the general population [10]. Qualified psychotherapists include mental health professionals such as mental health nurses, psychologists, clinical social workers, and psychiatrists [7].

### 1.3. Complementary and Alternative Medicine

Complementary and alternative medicine (CAM) is a rapidly growing field in Australian healthcare and research. CAM is utilized in conjunction with (complementary) or as a replacement for (alternative) standard health practices. Such growth is identified as a direct result of limited success with some conventional treatments for general psychopathology symptomology, as well as a progressive societal shift to more open mindsets [11].

There is a substantial amount of literature investigating reasons for clients' choice of alternative psychotherapy treatments [12–14]. Mercer [14] claimed individuals who engage in alternative treatments have difficulty thinking through pseudoscientific claims and are inclined to accept implausible arguments on faith. In contrast, Lee et al. [13] have identified that many clients of alternative therapist's dislike aspects of conventional treatment, in particular the perceived power imbalance, whereby some individuals report decreased locus of control. Such interpersonal aspects of treatment have particular significance with regard to psychotherapy, where there is a large negative societal stigma associated with mental illness.

Leach [11] conducted research to profile Australian attitudes to CAM, specifically measuring the differences between individuals who utilize CAM and nonusers in terms of predisposing factors, enabling factors, need factors, and personal health practices. Results indicated a clear distinction between CAM users and their nonusing counterparts within each of the factors investigated. More specifically, CAM consumers reported overall healthier lifestyle behaviours, notwithstanding their identified increased healthcare needs. The utilization of CAM in high-need populations may indicate inadequacy of the present healthcare system to address consumer needs. Furthermore, Reid et al. [15] investigated the utilization, perceptions, and factors associated with the use of complementary medicine (CM) by Australians. Results found a correlation between higher usage rates in females compared to males. Furthermore, females who utilized CM were more likely to be of middle-age, have higher education, and from an elevated socioeconomic stratum compared to their non-CM using female counterparts.

Importantly, there are ethical implications associated with the use of CAM, which are best understood through the four predominant bioethics principles: respect for autonomy, justice, beneficence and nonmaleficence [16]. Bioethics refers to ethics pertaining to life and how therapists purposely alter it; hence, it has particular significance in medicine, healthcare, and biomedical research. Autonomy is the right for an individual to make their own informed choice without external influence. Justice emphasizes fairness and equality among individuals. Beneficence refers to acting with the patient's best interest in mind, thus ensuring the risks involved do not outweigh the potential benefits of the treatment. Nonmaleficence aligns with a statement in the Hippocratic Oath; above all, do no harm.

Respecting each principle is not always straightforward [16]. Some therapies have the potential to cause harm, in which case nonmaleficence would infer that the harm needs to be considerably less than the benefits of the therapy. Moreover, in some circumstances, abiding by the principles of nonmaleficence and beneficence may require a decreased priority in respecting one's autonomy and vice versa. For example, a particular treatment may be potentially lifesaving; however, the patient who is in adequate health to make the decision chooses not to undergo the therapy. As such, the utilization of CAM often gives rise to ethical dilemmas as a result of heath professional and patient perceptions.

#### 1.3.1. Translational Gap

The translational gap refers to the time gap between new therapies having passed ethical standards and being conducted in practice, until they are considered standard care [17, 18]. The translational gap has been identified as approximately 17 years, with only 20% of new therapies accepted in mainstream treatment. The translational gap has direct relevance to alternative therapies, and specifically, equine-assisted psychotherapy (EAP) will be discussed in the following review.

### 1.4. Animal-Assisted Therapy

**Figure 1:**
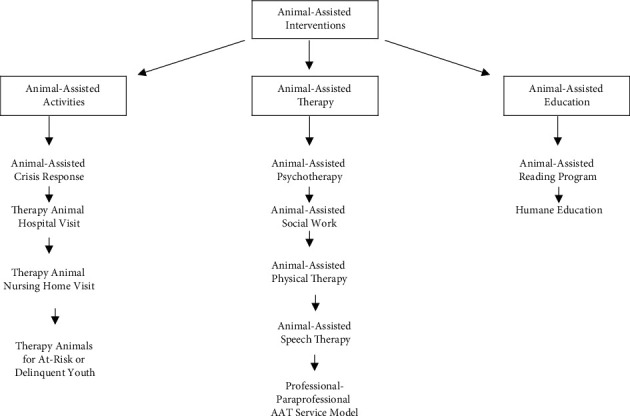
Fine's [19] spectrum of animal-assisted interventions.

Animal-assisted interventions (AAI) consist of goal-directed interventions aimed to stimulate improvement in one's cognitive, physical, social, and/or emotional functioning [19]. The intervention is provided by a specialized practitioner, who is assisted by a professionally trained animal-handler team. AAI is an umbrella term which covers a large spectrum of therapeutic interventions, as shown in Figure 1.

Animal-assisted therapy (AAT) is deliberately designed to integrate pedagogical, psychological, and social interventions with animals for individuals of all ages (children to senior citizens) who have cognitive, behavioural, or social-emotional difficulties or motoric disabilities [19]. AAT can be incorporated across the full mental health continuum of care, from prevention, early intervention, acute treatment to recovery. The development and advancement of contemporary society has been greatly influenced by the human-animal connection. Animals fulfil various roles in human lives such as companions, physical supports, coworkers, confidants, and cotherapists, as well as sources of food and clothing materials [20]. The field of human-animal interactions (HAIs) is progressively evolving to recognize the adept capabilities of animals to benefit mankind beyond their tradition role of beasts of burden. Interestingly, more than 60% of Australian pet owners deem their pet a member of the family [21]. Moreover, “pet parenting” behaviours have been adopted by pet owners, which resemble parent-child relationships, as such, it is evident that pets play an increasingly vital role in the lives of humans. Animals that often facilitate therapy include horses, dogs, cats, pigs, and birds. Importantly, each species possesses various characteristics, which when enhanced by targeted training offers a diverse range of assistance within the therapy setting [20]. Horses (equines) are an increasingly popular animal choice within therapy, as therapists become more aware of the extensive therapeutic enabling capabilities of the horse [22]. Horses are prey animals which heightens their sensitivity to their surroundings and encourages contact with their herd for validation, comfort, and reassurance [23].

### 1.5. Equine-Assisted Therapy

Equine-assisted therapy (EAT) broadly covers any type of treatment or therapy that comprises equine activities, interactions, or treatment [22]. Practices must be delivered by appropriately trained and accredited healthcare professionals and are regulated by healthcare laws. There are four subcategories which exist under the umbrella term EAT. They are equine-assisted: psychotherapy, physical therapy, occupational therapy, and speech therapy. In equine-assisted psychotherapy (EAP), the licensed practitioner utilizes the horse as a perceptive cofacilitator, and consequently, the horse is an active and valuable component of the therapy. EAP is the subcategory of EAT that will be investigated throughout the following study.

Horses are a prey animal and thus living in the present moment is essential for their survival [24]. They are extremely sensitive to their surroundings and have a strong ability to recognize and attend to nonverbal cues. Consequently, horses are sensitive to human emotions which facilitate many therapy settings [25, 26]. Horses sense and respond to incongruencies between individuals' actions and their underlying feelings. Specifically, horses connect and engage with clients when the individual is perceived by the horse to be in a state of physical and emotional congruence [23, 27]. Moreover, a unique opportunity for feedback and learning is presented to clients through the horses' ability to imitate their emotions in real time [28].

### 1.6. Equine-Assisted Psychotherapy

EAP is a collaborative process where a licensed mental health professional works alongside an equine specialist and therapy horse/s to address psychotherapeutic objectives [23]. Clients engage in on-ground activities with the horses, where their relative emotions, behaviours, thoughts, and feelings are discussed and further analyzed [29].

There are multiple EAP techniques with varying accreditations and associated literature. One commonly utilized association is the Equine-Assisted Growth and Learning Association (EAGALA) model of EAP. Within this model, practitioners provide feedback to clients, regarding identified behavioural patterns of the client and distinctive movements and discrepancies in the horses' behaviour (Thomas & Lytle, 2016). Moreover, horses are able to maintain more objective responses to human moods, accurately reflecting individual's emotional energy and serving as an effective means of transference learning [22, 23].

Horses have unique personalities, as do humans, in therapy settings, individuals may choose a therapy horse in which they can assimilate to [30]. Subsequently, throughout the therapy sessions, an individual often empathizes with their horse, which in turn helps the client to process experientially issues in their life and enables a heightened understanding of the individuals own interpersonal relationships. Additionally, although a horses' imposing size and their ability to induce fear may appear counter-intuitive, it offers clients the opportunity to build self-efficacy, overcome fear, and gain a sense of mastery [23].

The inclusion of the horse whether it be through active engagement or through observation, assists clients to self-explore their issues in a more creative manner, particularly with the utilization of strong metaphors. Compared to traditional talk therapy, EAP enables a reduced reliance upon verbal communication between client and therapist and an overall less intrusive engagement [23, 30]. Accordingly, EAP seems a suitable response to the aforementioned need for culturally sensitive therapies [31].

EAP has similar components to conventional therapy as practitioners set specific therapeutic objectives, guide the interactions with the client, and measure and evaluate overall progress [32]. EAP has been found to be a valuable complementary practice alongside conventional therapies, in the circumstance that the client and the practitioner have a clear understanding of the horse's purpose and the potential benefits [33]. Moreover, multiple studies have found that clients who do not have success with more conventional therapy respond well to EAP [29, 30].

Existing research within the field of EAP has consistently identified the following outcomes of EAP sessions: enhanced intuition, increased attention on the present, better adjustments to emotional changes, increased self-care and confidence, increased openness, improved social skills, healthier interpersonal relationships and communication, improved conflict resolution, successful boundary implementation, and improved psychological well-being [23, 29]. Moreover, enhanced self-reflection and a sense of trust are identified as direct results of the horses' presence; as distinct from humans, horses' responses are not socially conditioned [26].

Contemporary literature pertaining to EAP includes Earles et al.' [34] assessment of the effectiveness of equine-assisted therapy for anxiety and posttraumatic stress symptoms. The author specifies the term “therapy” rather than psychotherapy; however, upon evaluation, it is clear that they are investigating EAP and have used inappropriate terminology. This has been a consistent theme throughout multiple studies and may contribute to the lack of understanding in the clinical and general community populations. Despite the identified limitations of this study, results concluded that EAP effectively treats PTSD and other anxiety symptoms. Participants reported significant reductions in anxiety, depression, and trauma-related distress after their six-week EAP program.

More recently, Hallberg [22] performed a comprehensive literature review covering a broad range of therapeutic interactions with horses, as well as a specified focus on equine programs used in mental health. Overall, the reviews were cautiously hopeful regarding the efficacy of EAP; however, there was no methodological rigor, and thus, the results could not conclusively attest to the benefits of EAP. More specifically, 24 published research articles on the effectiveness of EAP on autism were reviewed, with findings showing benefits including reduced overall autism symptoms and increased self-regulation and social skills. Furthermore, of the 10 studies investigating general mental illness and EAP, 9 found enhanced well-being and self-esteem and reduced depression levels.

The identification of target populations who may benefit most from EAP is also an emerging research area. Bennett and Woodman [31] published a literature review on the potential of equine-assisted psychotherapy for treating trauma in Australian Aboriginal people. Results found EAP to be an effective treatment method for indigenous populations due to its flexibility and applicability to cultural diversity. The overall body of literature concerning the efficacy of EAP is best described as cautiously hopeful, as although the existing outcomes appear promising, the current state of literature has an insufficient amount of evidenced based research and thus precludes research from determining a definitive conclusion of the efficacity of EAP [12].

### 1.7. Perceptions of EAP

Wilson et al. [35] conducted an Australian-based qualitative study to investigate EAP practitioner perspectives on the biopsychosocial benefits and therapeutic outcomes of EAP for adolescents experiencing depression and/or anxiety. Practitioners consistently identified and contributed the following client' outcomes as direct benefits of EAP: increased confidence, self-esteem, and assertiveness, as well as the decrease in undesirable behaviours. Prior experience with horses was addressed as a potential mediator in the effectiveness of EAP. Within the study, therapists identified the consistent theme that previous horse experience delayed the effectiveness of the therapy, as individuals displayed difficulties embracing the metaphors. Specifically, therapists reported that participants with previous horse experience had difficulty processing the horse's reactions as mirroring reflections of their own behaviours. However, therapist consensus maintained that EAP outcomes would not be affected by a client's previous experiences with horses. Finally, enhancing EAP as a treatment modality was addressed by EAP practitioners. Each therapist identified a lack of understanding in the wider community with what specifically constitutes EAP and how it works. Furthermore, it was of common perspective that regardless of health professional's knowledge of EAP, they felt an ethical dilemma in referring clients with its limited evidence base. Overall, EAP practitioners recognized the need for a greater evidence base in the EAP field to enhance the knowledge of Australia's wider community.

Furthermore, perceived benefits of EAP have been assessed in older individuals who have participated in the therapy. Lee et al. [13] utilized a mixed method study design with a concurrent triangulation approach to assess older adults' perceived benefits of EAP and more specifically to determine whether or not older adults with functional and/or cognitive impairment found meaning and purpose in their interactions with horses. Results indicated that older individuals with functional or cognitive impairment were able to meaningfully engage in the EAGALA model of EAP and find purpose from the experience. Importantly, participants' perceived benefits surpassed the human-horse interaction to the outdoor setting, as well as an increased level of social interactions through reminiscence and positive influences from peers. Moreover, older adults felt reduced stigma within EAP compared to traditional talk therapy.

There is little contemporary literature pertaining to individuals' perceptions of EAP, and the existing content is limited to populations within the EAP field, either individuals who have practiced (health professionals) or participated in (clients) EAP. Equine-assisted psychotherapists presume that the wider community has a lack of understanding about EAP, which subsequently exists as a barrier to the recognition of and acceptance of EAP as a valid therapeutic intervention. Importantly, few studies have precisely identified perceptions of EAP, of the general community and health professionals outside the EAP field; thus, literature cannot conclusively attest to the causation of minimal referrals to EAP and whether it is a result of lack of knowledge or acceptance from either health professionals or clients.

#### 1.7.1. Predictors of Accepting Perceptions of EAP

Predictors of accepting perceptions of EAP have not been directly assessed within the literature; however, predictors of accepting perceptions of CAM and reasons for utilizing AAT and EAT have been identified [11, 36]. Subsequently, potential predictors for accepting perceptions of EAP are established based on their indirect relevance. High openness to experience is a character trait identified as a significant predictor for higher levels of utilization and acceptance of CAM [37]. Specifically, such individuals appear overall more supportive of new natural and more holistic merging healthcare practices. Lower levels of social support are suggested to be a predictor for acceptance and utilization of AAT [36]. Literature suggests that many individuals find it easier to interact with and feel more open around domesticated animals, particularly for individuals with reduced perceived social support.

Furthermore, Irvine [36] found that individuals who owned a pet were more likely to engage with animal-related interventions, which were identified as a reflection of their comfort with and around animals. Similarly, previous horse experience has been identified as a predictor for utilization of and acceptance of EAT [22]. Such individuals have more accurate conceptions of the adept healing capabilities of the horse, beyond those of other domesticated animals.

Individuals from rural locations are also identified as being more likely to utilize and/or accept EAT, as a result of their familiarity with horses through their surroundings [38, 39]. Rural individuals are more likely to own a horse or have some previous horse experience compared to their metropolitan counterparts and subsequently have a better understanding of horses healing potential.

### 1.8. The Current Study

Contemporary literature in the field of EAP, whilst limited, consistently indicates promising outcomes for the treatment for general psychopathology symptomology [22, 31]. However, EAP remains an underutilized intervention in Australia. One of the gaps within the literature is the reason more clients do not exploit the therapy. Although there is no empirical evidence to support the claim, many equine-assisted psychotherapists presume it relates to a lack of general understanding of what constitutes EAP. There are limited studies assessing perceptions of EAP, and existing content is constrained to perceptions of individuals within the field.

Therefore, the current Australian-based quantitative survey aimed to determine the level of knowledge about and general acceptance of EAP as treatment for general psychopathology symptomology. The sample addressed in this survey included general community members and health professionals 18 years or older, thus being one of the first studies to address perceptions of individuals outside the EAP field. To guide the study, the following research questions were investigated. Is there a disparity between community members and health professionals' perceptions of EAP and how can accepting perceptions of EAP be best explained within each group. Importantly, these predictors were proposed because of their relevance to each group, that is, community members may participate in the therapy, whilst health professionals may refer clients, but not participate in the therapy. Additionally, a limitation in contemporary literature is the inconsistent and invalid use of proper terminology surrounding EAP. To address this issues, clear and concise definitions and distinctions were made for and between AAT, EAT, and EAP in the present study.

It was anticipated that results of the present study may assist in the expansion of EAP into mainstream therapy from its current position within the translational gap. The present study aimed to develop understanding of whether unaccepting perceptions were evident across both groups (community members; clients or health professionals; referring clients). Subsequently, profiling each group, through their predictors of accepting perceptions of EAP, may now guide proponents in establishing more appropriate promotional work and research, as well as identify target populations who may benefit most from EAP.

#### 1.8.1. Hypotheses

It was first hypothesized that community members would be significantly more accepting of EAP as a treatment for general psychopathology symptomatology, compared to health professionals, as indicated by higher scores on the Behaviour Intervention Rating Scale (BIRS). Second, it was hypothesized that higher levels of openness would significantly predict more accepting perceptions of EAP, in both general community members and health professionals (as indicated by the BIRS measure). Third, it was hypothesized that rural locality would significantly predict more accepting perceptions of EAP, in both community members and health professionals. Fourth, it was hypothesized that that lower levels of perceived social support as measured by the Multidimensional Scale of Perceived Social Support (MSPSS) would significantly predict more accepting perceptions of EAP in community members. The final hypothesis proposed that previous horse experience would significantly predict more accepting perceptions of EAP in community members.

## 2. Methods

### 2.1. Participants

Inclusion criteria required individuals to be 18 years of age or older and have Australian citizenship. Participation encompassed both genders, and individuals were drawn from the general community and health professional (i.e., psychologists, counsellor, and general practitioner) populations/backgrounds. To be representative of the Australian population over 18 years, with a confidence level of 95% and a margin of error of 5%, the sample was required to be 385 subjects. Although 240 participants (183 community members and 57 health professionals) were initially recruited, the final sample of the present study consisted of 184 participants, and thus, this needs to be acknowledged against the predicted sample size. Specifically, 129 community members (97 females and 32 males) and 55 health professionals (36 females and 19 males), after application of the exclusion criteria and data cleaning to meet statistical assumption checks. The community member sample had an age range of 18–65 years (M = 33.82, SD = 14.14), and the health professional sample had an age range of 21–68 years (M = 44.07, SD = 12.97). For the HMR, 10 participants were required per predictor [40]. To accommodate this, nationality was excluded as a predictor across both groups, and education level was collapsed into the categories of bachelor's degree and lower vs. master's degree and higher within the community members sample (this variable was not a measure of interest in health professional sample). A detailed breakdown of participant demographic information, across each group, is given in Table 1.

### 2.2. Materials

An online (anonymous) questionnaire package was developed for the research project which included three scales and a set of demographic questions. The scales Behaviour Intervention Rating Scale, the Mini-Marker, and the Multidimensional Scale of Perceived Social Support were presented, respectively. The demographic questions encompassed age, gender, and status as required for the study (health professional or general community member), any previous psychological diagnoses, marital status, education level, socioeconomic status based on annual income, residential situation (rural or metropolitan), previous experience with therapy or horses, and whether of first nations heritage (nationality). Two materials were required to complete this survey: a digital device such as a computer, laptop, or mobile phone and a relevant social media account such as Instagram, LinkedIn, or Facebook.

#### 2.2.1. Behaviour Intervention Rating Scale

The Behaviour Intervention Rating Scale (BIRS) is a modification and extension of the Intervention Rating Profile (IRP-15; [41]). The IRP-15 is a single-factor scale with 15-items, utilized to assess treatment acceptability. The IRP-15 demonstrates high internal consistency reliability, as measured by a Cronbach's alpha of 0.98. The BIRS is a 24-item instrument, originally developed to measure teachers' perceptions of treatment acceptability and treatment effectiveness. Participants are provided information about a child with a classroom problem and a description of a proposed intervention for that problem. Participants are then required to evaluate the intervention using a 5-point Likert scale ranging from 1 (strongly disagree) to 5 (strongly agree). Higher total score is indicative of higher treatment acceptability. An example statement is “The child's behaviour will remain at an improved level even after the intervention is discontinued.” There are no negatively worded items in this scale. To determine the BIRS's concurrent validity, Elliot and [42] correlated BIRS components against the evaluation factor of the semantic differential (SD) rating scale [43]. There was a high correlation between the evaluation factor of the SD and the BIRS acceptability factor (0.78) and the BIRS effectiveness factor (0.67), as well as a moderate correlation between the evaluation factor of the SD and the BIRS time factor (0.52). To determine the BIRS reliability, coefficient alphas were used. The BIRS, in full, yielded an alpha of 0.97, while the three components, acceptability, effectiveness, and time, yielded alphas of 0.97, 0.92, and 0.87, respectively. Therefore, these results are supportive of a reliable and valid scale.

Although the BIRS was established from a behavioural orientation, its application is not limited to behavioural interventions. The BIRS is consistently demonstrated to be an efficacious measure of perceptions of treatment acceptability and treatment effectiveness. For the purpose of the present study, the scale was adapted to EAP. Specifically, participants were provided with a scenario regarding a client with general psychopathology symptomatology and a description of a corresponding equine-assisted psychotherapy session. Individuals were asked to evaluate the intervention by indicating how much they agreed or disagreed with each statement. An example statement was “Equine-assisted psychotherapy would be an acceptable intervention for the client's potential disorder.” Responses were recorded on a 5-point Likert scale ranging from 1 (strongly disagree) to 5 (strongly agree). Higher total score indicated higher acceptability for the equine-assisted psychotherapy intervention. There were no negatively worded questions. Within the current study, the adapted BIRS yielded a Cronbach's alpha coefficient of 0.95, indicating high reliability.

#### 2.2.2. The Mini-Marker

The Mini-Marker (MMPS; [44]) was employed in the current study as a brief and efficient tool for evaluating participants' level of openness. The MMPS is a short-form subset of Goldberg's 100 adjective Unipolar Big-Five Markers. The MMPS has 40 adjectives, each of which loads onto one of the five factors, extraversion, agreeableness, conscientiousness, emotional stability, and openness. The MMPS was developed to address the timely nature of Goldberg's original scale, as well as the language and comprehension difficulties, particularly in multinational samples [44, 45].

Each of the big-five personality traits has eight markers. Negative and positive poles of each factor are included within the scale [44]. Openness is the fifth factor, comprised of the following markers: imaginative, philosophical, creative, deep, complex, intellectual, uncreative, and unintellectual. Participants are required to make a contemporaneous response, to each marker, using a 9-point Likert scale ranging from 1 (extremely inaccurate) to 9 (extremely accurate). High total scores of each of the five factors indicate that the participant possesses higher levels of that facet. For openness, unintellectual and uncreative were reverse scored.

The MMPS is widely accepted as a measurement scale, based on its quality of psychometric properties [44–46]. High reliability is demonstrated by each factor having high internal consistency: extraversion (*α* = 0.83), agreeableness (*α* = 0.81), conscientiousness (*α* = 0.83), emotional stability (*α* = 0.78), and openness (*α* = 0.78; [44, 47]. Convergent validity has been established between the MMPS and Mooradian and Nezlek's [48] measure of personality, where the shared variance of each of the measure's subscales ranged 25–50%. Of note, the MMPS has lower interitem correlations compared to Goldberg's original full scale [44, 47]. Overall, the scale has sound psychometrics for use in the current study.

#### 2.2.3. The Multidimensional Scale of Perceived Social Support

The Multidimensional Scale of Perceived Social Support (MSPSS) is a 12-item measure of subjectively assessed social support from family, friends, and significant others. An example item is “My family really tries to help me.” Response choices for each item range from 1 (very strongly disagree) to 7 (very strongly agree). Item responses are totalled to produce a total item score for the scale, ranging from 12 to 84. Higher scores are indicative of greater levels of perceived social support for the respondent. There are no negatively worded items. The MSPSS has proven to be psychometrically sound in diverse samples, particularly through its good internal reliability, test-retest reliability, and concurrent validity [49].

MSPSS has demonstrated high internal consistency, within the total scale (*α* = 0.90), as well as each of the subscales, significant others (*α* = 0.86), family (*α* = 0.89), and friends (*α* = 0.87; [50]. The MPSS has also shown strong temporal stability, with a minimum kappa value of 0.67 in test-retest reliability analyses [51]. Concurrent validity of the MSPSS has been assessed with the State Trait Anxiety Inventory (STAI), the Thai Depression Inventory (TDI), and the Rosenberg Self-Esteem Scale (RSE). The MSPSS has a negative correlation with the STAI (*r* = 0.20, *p*=0.004) and TDI (*r* = −0.19, *p*=0.007). Conversely, it has a positively correlation with RSE (*r* = 0.33, *p* < 0.001). Combined, these results indicate acceptable external validity. The reliability of the MSPSS was assessed within the current study and yielded a Cronbach's alpha of 0.94, indicating high reliability.

### 2.3. Procedure

Prior to commencing the research, ethical approval was obtained from the Bond University Human Research Ethics Committee (approval: KG00153). Participants were recruited across social media platforms (e.g., Facebook, LinkedIn, and Instagram) where the Qualtrics survey was shared by the researcher. Snowballing was utilized for further dispersal. All participants were required to be Australian citizens, at least 18 years of age, and could not participate if they were an equine-assisted therapist. Upon opening the survey, participants were shown an explanatory statement, which provided the rationale for the study, time needed for survey completion (approximately 25 minutes), and subsequent ethical protocols of the study. Participation was voluntary, no incentives were provided for participation, and participants could withdraw at any time without penalty. Data retrieved were anonymous and confidential, and there were no follow-up protocols. After the explanatory statement, participants were required to give their consent. If consent was provided, the survey continues; however, if consent was not obtained, the survey would end. Data were retrieved through self-report questionnaires utilizing Qualtrics, a secure web-based system. Subsequently, responses were downloaded directly to SPSS for data screening, cleaning, and analysis.

## 3. Results

### 3.1. Data Diagnostics

Prior to running the analyses, the data were screened for data entry errors and criteria violations using frequency statistics as well as ocular discrepancy. There were 240 participants, 183 community members, and 57 health professionals. Incomplete/missing data comprised 15.4% (37 cases) for community members and 8% (19 cases) for health professionals, missing at random. Tabachnick and Fidell [52] indicate that over 5% missing data are considered a threat and skews the data; thus, the incomplete cases in the community members sample were removed without hesitation due to the large sample size. In contrast, mean imputation was utilized for the incomplete data in health professionals to ensure the sample was sufficient for further analysis. Mean imputation was suitable in this scenario as the data were missing at random. Although this method ensured a larger sample size, it may lead to an underestimate of the errors; thus, results were interpreted with caution [53].

A visual inspection of box and whisker plots identified ten univariate outliers (eight community members and two health professionals), prompting an examination of *z*-scores. According to Tabachnick and Fidell [52], *z*-scores that exceed ±3.29 are extreme values. Both outliers in the health professional sample as well as two in the community member sample exceeded +3.29. As such, these scores were removed from the analysis (*N* = 4). Hence, the sample was reduced to 199 participants (55 health professionals and 144 community members) after initial data screening. After addressing criteria violations, missing data, and univariate outliers, the statistical assumptions of the two-way independent *t*-test and HMR's were separately assessed.

### 3.2. Two-Way Independent *t*-test Assumptions

A two-tailed, independent samples *t*-test, with an alpha level of 0.05, was conducted to determine if there was significant difference of perceptions of EAP (DV) in community members (IV_1_) compared to health professionals (IV_2_). The two methodological assumptions, scale of measurement and independence, were met. Assumptions of normality and homogeneity of variance were checked. Normality was confirmed for both groups via visual inspection of Q-Q plots, Shapiro–Wilks statistic (*p*=0.09), and skewness and kurtosis values which were all within the range. Notably, the sample size was smaller than intended (*N* = 199); however, 80% power could be obtained with a large effect. Nonparametric tests are often utilized when normality is violated and/or there is a small sample size; however, they do not provide reliable results when the samples have differing amounts of variability [54]. Therefore, the parametric procedure was chosen instead. Levene's statistics was significant (*p*=0.02), and thus, the assumption of homogeneity of variance was violated. However, SPSS is robust against this violation and provides a *t*-test for equal variance not assumed (Welch's *t*-test), which was utilized.

### 3.3. Main Analysis

#### 3.3.1. Difference between Community Members and Health Professionals EAP Perceptions

The *t-*test for equal variances not assumed was statistically significant at *α* = 0.05. Thus, the null hypothesis was rejected, and it was concluded that community members were significantly more accepting of EAP as treatment for general psychopathology symptomology than health professionals, *t* (76) = −5.72, *p* < 0.001. Means and standard deviations are given in Table 2. The magnitude of the differences in the means (−14.23, 95% CI*:* −19.18 to −9.27) was large (Cohen's *d* = 1.05). However, these results need to be interpreted with caution as the sample size is small, and this was a preliminary investigation.

### 3.4. Community Members HMR

#### 3.4.1. Assumptions

To estimate the proportion of variance in community members' perceptions of EAP (continuous) that can be accounted for by perceived social support (continuous), openness to experience (continuous), previous experience with horses (dichotomous), pet ownership (dichotomous), and previous engagement in equine-therapy (dichotomous), a HMR was performed. An alpha level of 0.05 was used for all statistical analysis, unless otherwise stated.

Prior to interpreting the results of the HMR, a number of assumptions were tested, and checks were performed. An examination of Mahalanobis' distance values identified 15 multivariate outliers that exceeded *α* = 0.001. Such outliers were deleted to ensure the dataset was not skewed. It was subsequently acknowledged that the sample size (*N* = 129) was sufficient, as 10 observations are required per prediction, requiring a minimal sample size of 110 participants [55].

Normality was assessed via a visual inspection of Q-Q plots which indicated that each variable was approximately normally distributed, except PSS which was slightly negatively skewed. To further examine this, skewness (*N* < −1 = negatively skewed) or (*N* > 1 = positively skewed) and kurtosis (*N* ≊ ± 3) were assessed. All were within range, except PSS was slightly negatively skewed (−1.12). Thus, square root and logarithmic transformations were applied to PSS. To test whether nonnormality had an impact on the results, transformed data were compared to nontransformed data. The transformed data did not improve normality. Since these distributions (higher PSS scores) could be expected in the current community sample, Tabachnik and Fidell's [52] indicate that mild departures from normality in HMR's are not of concern, and the nontransformed data were utilized for further analysis.

Independence of observation was assumed through Durbin-Watson statistic (2.22). Relatively low variance inflation factors (VIF) values (1.35) for all predictors in the final regression model indicated that multicollinearity would not interfere with the ability to interpret the outcome of the HMR. Finally, visual inspection of the scatterplot of standardized residuals against standardized predicted values and the normal probability plot of standardized residuals indicated that the assumptions of linearity, homoscedasticity, and normality of residuals were met.

### 3.5. Predictors of Community Members Perceptions of EAP

#### 3.5.1. Preliminary Analysis

Pearson's correlations (*r*) were utilized for analysis of the direction and effect size of the relationship between each independent variable on the criterion variable perceptions of EAP (M = 96.38; SD = 11.74). A small significant positive correlation was found between openness (M = 49.86; SD = 9.72) and perceptions of EAP (*r* *=* 0.18, *p* < 0.005), indicating that as openness increases, perceptions of EAP also increase. There were no other statistically significant correlations, as given in Table 3.

#### 3.5.2. Main Analysis

A six-stage HMR was employed with community member's perceptions of EAP as the criterion variable. Step one of the HMR is control for demographics such as age, gender, SES, marital status, education level, and clinical psychopathology diagnosis. These demographics accounted for a nonsignificant 6% of the variance in perceptions of EAP, *R*^2^ = 0.06, *F* (6, 121) = 1.29, *p*=0.273. On step two, perceived social support was added to the regression equation and accounted for an additional nonsignificant 2.5% of the variance in perceptions of EAP, ∆*R*^2^ = 0.03, ∆*F* (1, 120) = 3.28, *p*=0.073. On step three, openness to experience was added to the regression equation and accounted for an additional significant 3.1% of the variance in perceptions of EAP, ∆*R*^2^ = 0.03, ∆*F* (1, 119) = 4.27, *p*=0.041. On step four, pet ownership was added to the regression equation and accounted for an additional nonsignificant 1.9% of the variance in perceptions of EAP, ∆*R*^2^ = 0.02, ∆*F* (1, 117) = 2.50, *p*=0.177. On step five, previous horse experience was added to the regression equation and accounted for an additional significant 3% of the variance in perceptions of EAP, ∆*R*^2^ = 0.03, ∆*F* (1, 116) = 4.24, *p*=0.042. On step six, location was added to the regression equation and accounted for an additional nonsignificant 2.2% of the variance in perceptions of EAP, ∆*R*^2^ = 0.02, ∆*F* (1, 115) = 3.06, *p*=0.083.

The full model of demographics, perceived social support, openness, pet ownership, previous horse experience, and location (model 6) accounted for a statistically significant 18.7% of variance in community members' perceptions of EAP significant, *R*^2^ = 0.19, *F* (11, 127) = 2.42, *p*=0.010; adjusted *R*^2^ = 0.109. By Cohen's [56] conventions, a combined effect of this magnitude can be considered “small” (*f*^2^ = 0.04). Once the final model had been constructed, the only two significant variables were perceived social support and openness. Specifically, perceived social support was a significant positive predictor of accepting perceptions of EAP in community members. For every one standard deviation increase in perceived social support, there was a 0.23, 95% CI (0.06–0.52) standard deviation increase in accepting perceptions of EAP. Openness was also a significant positive predictor of accepting perceptions of EAP in community members. For every one standard deviation increase in perceived social support, there was a 0.18, 95% CI (0.01–0.44) standard deviation increase in accepting perceptions of EAP.

Unstandardized (*B*) and standardized (*β*) regression coefficients and squared semipartial (or “part”) correlations (sr^2^) for each predictor on each step of the HMR and associated 95% confidence intervals are given in Table 4.

Moreover, whilst social support (sr^2^ = 0.04) and openness (sr^2^ = 0.03) remained significant at the final step, it is worth noting that previous horse experience demonstrated a significant positive trend when added at step four. However, the amount of variance it contributed was not significant in the final model, resulting in its final nonsignificance.

### 3.6. Health Professionals HMR

#### 3.6.1. Assumptions

To estimate the proportion of variance in health professionals' perceptions of EAP (continuous) that can be accounted for by perceived social support (continuous), openness to experience (continuous), previous experience with horses (dichotomous), pet ownership (dichotomous), and previous engagement in equine-therapy (dichotomous), a HMR was performed. Unless otherwise stated, an alpha level of 0.05 was used for all statistical analysis.

Prior to interpreting the results of the HMR, a number of assumptions were tested. An examination of Mahalanobis' distance indicated that there were no multivariate outliers that exceeded *α* = 0.001; thus, the assumption was met. It was subsequently acknowledged that the sample size (*N* = 55) was sufficient in accordance with statistics solutions [40], which require 10 observations per predictor, requiring a minimal sample size of 50 participants for this analysis.

Normality was assessed via a visual inspection of Q-Q plots which indicated that each variable was approximately normally distributed, except perceived social support which was slightly negatively skewed. To further examine this, skewness (*N* < −1 = negatively skewed) or (*N* > 1 = positively skewed) and kurtosis (*N* ≊  ± 3) were assessed. All were within range, except perceived social support which was slightly negatively skewed (−1.42). Thus, square root and logarithmic transformations were applied to perceived social support. To test whether nonnormality had an impact on the results, the transformed data were compared to the nontransformed data. The transformed data did not improve normality. Since these distributions could be expected in the current health professional sample, which is further addressed in the discussion, Tabachnik and Fidell's [52] indicate that mild departures from normality in HMR's are not of concern, and the nontransformed data were utilized for further analysis.

The Durbin-Watson statistic (1.59) indicated an absence of autocorrelation; thus, the independence of observation assumption was met. Relatively low variance inflation factors (VIF) values (1.08) for all predictors in the final regression model indicated that multicollinearity would not interfere with the ability to interpret the outcome of the HMR. Finally, visual inspection of the scatterplot of standardized residuals against standardized predicted values and the normal probability plot of standardized residuals indicated that the assumptions of linearity, homoscedasticity, and normality of residuals were met.

### 3.7. Predictors of Health Professionals Perceptions of EAP

#### 3.7.1. Preliminary Analysis

Pearson's correlation (*r*) was utilized for analysis of the direction and effect size of the relationship between each independent variable on the criterion variable, perceptions of EAP (M = 82.62; SD = 16.86). A small significant negative correlation was found between location (M = 1.73; SD = 0.45) and perceptions of EAP (*r* *=* −0.29, *p* < 0.005). There were no other statistically significant correlations, as given in Table 5.

#### 3.7.2. Main Analysis

A four-stage HMR was employed with perceptions of EAP as the criterion variable. Step one of the HMR is control for demographics such as age and gender. Combined, age and gender accounted for a nonsignificant 0.6% of the variance in perceptions of EAP, *R*^2^ = 0.06, *F* (2, 52) = 0.15, *p*=0.860. On step two, previous horse experience was added to the regression equation and accounted for an additional nonsignificant 0.1% of the variance in perceptions of EAP, ∆*R*^2^ = 0.00, ∆*F* (1, 51) = 0.06, *p*=0.813. On step three, openness to experience was added to the regression equation and accounted for no additional variance in perceptions of EAP, ∆*R*^2^ = 0.00, ∆*F* (1, 50) = 0.02, *p*=0.889. On step four, location was added to the regression equation and accounted for an additional significant 9.7% of the variance in perceptions of EAP, ∆*R*^2^ = 0.09, ∆*F* (1, 49) = 4.85, *p*=0.032.

The full model of age, gender, previous horse experience, openness, and location (model 4) accounted for nonsignificant 9.7% of variance in health professionals' perceptions of EAP, *R*^2^ = 0.10 *F* (5, 54) = 1.04, *p*=0.401. Unstandardized (*B*) and standardized (*β*) regression coefficients and squared semipartial (or “part”) correlations (sr^2^) for each predictor on each step of the HMR and associated 95% confidence intervals are given in Table 6. Location (sr^2^ = 0.09) is the only significant predictor of health professionals' perceptions of EAP in the final regression.

#### 3.7.3. Follow-Up Analysis

Location was a significant negative predictor of EAP perceptions. As location was coded to represent rural as 0 and urban as 1, this trend suggests the urban participants have poorer perceptions of EAP than their rural counterparts. Given the categorical nature of the variables, this trend is more clearly illustrated by use of a follow-up *t*-test.

Levene's statistics was nonsignificant (*p*=0.20), and thus, the assumption of homogeneity of variance was met. The *t-*test for equal variances assumed was statistically significant at *α* = 0.05. Thus, the null hypothesis was rejected, and it is concluded that rural health professionals were significantly more accepting of EAP as treatment for general psychopathology symptomology than metropolitan health professionals, *t* (53) = 2.21, *p* < 0.05. Means and standard deviations are given in Table 7. The magnitude of the differences in the means (10.88, 95% CI: 0.99–20.77) was large (Cohen's *d* = 1.54).

## 4. Discussion

The present study aimed to determine the level of knowledge about and general acceptance of EAP as a treatment for general psychopathology symptomology within community members and health professionals. However, these results need to be interpreted with caution as the sample size is small, and this was a preliminary investigation. The two following research questions were addressed, respectively. Is there a disparity between community members and health professionals' perceptions of EAP, and how can accepting perceptions of EAP be best explained within each group. The general communities are viewed as potential treatment recipients and health professionals as individuals who are in a position to refer clients to the therapy. Predictor variables should therefore be uniquely taken into account for each group. The current study did not investigate the predictor variables for each group as a direct comparison, but rather as complementary to one another.

Five hypotheses were put forward to guide the study and were addressed utilizing the independent *t*-test and two HMR's. It was first hypothesized that community members would be significantly more accepting of EAP as a treatment for general psychopathology symptomatology, compared to health professionals (as indicated by higher scores on the BIRS). This hypothesis was met, which is consistent with existing literature that indicates health professionals are sceptical of and lack general acceptance for EAP [35]. Lee et al. [13] state that this scepticism is not a result of professional's valid assessment of the therapy, but instead caused by their minimal knowledge of what specifically constitutes EAP. Health professional's lack of general awareness of EAP as identified within the current study has potential negative ramifications on the general Australian population. Male, rural and indigenous Australians have a greater need for more culturally sensitive therapeutic practices [31]. EAP has the potential to facilitate mitigation of this need, through its reduced reliance upon effective communication between the client and practitioner, as well as the amelioration of stigma. However, health professionals appear to be reluctant to promote this transition. Accordingly, health professionals may be compromising their patients' rights to autonomy and justice. Future research needs to assess the degree to which bioethical principles are respected by health professionals in relation to EAP in a larger sample.

The second hypothesis proposed that higher levels of openness would significantly predict more accepting perceptions of EAP, in both general community members and health professionals (as indicated by the BIRS measure). This was partially met, as openness as a predictor was only significant within community members. Existing literature suggests openness as a predictor for increased acceptance and utilization of CAM, which stems from the individual's general openness to try new things and a reduced focus on traditional practices [37]. Openness may not be a significant predictor variable within the health professional cohort due to their professional responsibility to observe the bioethical principle of nonmaleficence. This reluctance to explore and adopt emerging therapies may be compounded by the perceived need to conform with traditional therapies, which have more consolidated evidence base. Moreover, previous literature did not discriminate between health professionals and community members in terms of openness predicting acceptance of EAP.

The third hypothesis indicated that rural locality would significantly predict more accepting perceptions of EAP, in both community members and health professionals. This was partially met as rural locality was only a significant predictor for health professionals. This result aligns with contemporary literature, which suggests that rural health professionals are directly and indirectly exposed to the potential benefits of EAP, through their surroundings and interactions with their client base [38, 39]. Interestingly, rural locality was not a significant predictor within the community members population. Although this finding is not consistent with existing literature, it suggests that there are other more immediate factors influencing an individual's perception of EAP. Notably, whilst the sample size was adequate across both community and health professional groups, there was an unequal distribution across the location demographic (rural and metropolitan). This has the potential to either inflate or conceal the varying trends between the groups; hence, these results should be generalized with caution.

The fourth hypothesis stated that lower levels of perceived social support, as measured by the MSPSS, would significantly predict more accepting perceptions of EAP in community members. This hypothesis was not met, and conversely, the results found higher levels of perceived social support to be a significant predictor of accepting perceptions of EAP in community members. This hypothesis was originally formed in alignment with Irvine [36] who indicates that individuals with lower levels of social support are most likely to benefit from EAP, as a result of its reduced reliance upon direct interpersonal communication and interaction with the practitioner. In contrast, results from the current study indicate that individuals who have higher levels of social support and understand the importance of interpersonal relationships see the benefits of fostering EAP. Notably, perceived social support scores were negatively skewed within the current sample, which may be a reflection of the current 2020 COVID-19 pandemic climate, whereby social media have been encouraging increased social support measures, during isolative-inducing restrictions.

The final hypothesis indicated that previous horse experience would significantly predict more accepting perceptions of EAP in community members. This hypothesis was not directly met, as previous horse experience was not a significant predictor within the final model of the regression. Although previous horse experience was not significant within the final model, it did show some significant contribution in earlier versions of the model for community members. While not found in this study, the trend would suggest that previous horse experience does contribute to accepting perceptions of EAP. Future research may utilize previous horse experience as a predictor of accepting perceptions of EAP; however, it should measure multiple aspects including type of horse interaction, whether they own a horse, and hours per week spent with horses to develop a more holistic understanding of how, and the degree to which, previous horse experience did contribute to accepting perceptions of EAP.

### 4.1. Limitations

Data collection in this study was via online means and participants voluntarily engaged, thus potentially threatening the validity of the research. It is acknowledged that significant differences have been reported when comparing results obtained through the use of passive recruitment surveys such as ours with purposive probability sampling, presumably due to sampling biases [57–59]. Future studies could investigate perceptions through the focus group or face-to-face mean in order to avoid sampling biases and passive recruitment issues. An ethical risk, also associated with the present study, is the potential unintended endorsement of a treatment technique which has limited evidence base. The existing literature on EAP consistently indicates that EAP is an effective treatment for mild psychological disorders across diverse samples; however, more research is needed to consolidate this. To minimise any public misconceptions, the study repeatedly reinforced EAP as a complementary, not alternative treatment. Moreover, the underpowered and unequal sample sizes posed potential statistical limitations, and thus, results were interpreted with caution. Furthermore, there was inadequate representation of first nation respondents to the survey; therefore, to ensure a valid statistical analysis, this category was collapsed. Future research should seek to address an adequate first nation sample.

### 4.2. Conclusions

Despite the limitations raised, the current study is one of the first to address perceptions of EAP as a treatment for general psychopathology symptomology of individuals outside the EAP field. It is also the first preliminary investigation of the disparities between community members and health professionals' perceptions of EAP. The results obtained from this study have tentatively identified health professionals as having a lower acceptance of EAP. Therefore, this group may benefit from the promotion of EAP through awareness and educational resources.

Further research needs to be conducted into the predictors of accepting perceptions of EAP in health professionals, as well as targeting a larger health professional sample size, inclusive of more rural practitioners. Importantly, previous horse experience as a potential predictor of acceptance of EAP should be examined in more detail to develop a more holistic understanding. Finally, future research should assess the degree to which bioethical principles are respected by health professionals in relation to EAP.

## Figures and Tables

**Table 1:** Distribution of participant characteristics by group (community member/health professional).

Participant demographics	Community members, *n* (%)	Health professionals, *n* (%)	Total, *N* (%)
Gender			
Male	32 (24.8)	19 (34.5)	51 (27.7)
Female	97 (75.2)	36 (65.5)	133 (72.3)

Age (years)			
18–40	87 (67.4)	23 (41.8)	110 (59.8)
41–59	37 (28.7)	25 (45.5)	62 (33.7)
60+	5 (3.9)	7 (12.7)	12 (6.5)

Highest level of education			
High school or equivalent	60 (46.5)	0	60 (32.6)
Vocational/technical college	18 (14)	0	18 (9.8)
Bachelor's degree	30 (23.3)	13 (23.7)	43 (23.4)
Master's degree	8 (6.2)	27 (49.1)	35 (19)
Doctoral degree	1 (0.8)	9 (16.4)	10 (5.4)
Others	12 (8.3)	6 (10.9)	18 (9.8)

Location			
Rural	96 (74.4)	15 (27.3)	111 (60.3)
Metropolitan	33 (25.6)	40 (72.7)	73 (39.7)

Households total annual income			
$0–$40000	23 (17.8)	6 (10.9)	29 (15.8)
$40001–$60000	25 (19.4)	1 (1.8)	26 (14.1)
$60001–$80000	24 (18.6)	3 (5.5)	27 (14.7)
$80001–$100000	24 (18.6)	11 (20)	35 (19)
$100001+	33 (25.6)	34 (61.8)	67 (36.4)

Marital status			
Divorced	7 (5.4)	3 (5.5)	10 (5.4)
Living with another	37 (28.7)	13 (23.6)	50 (27.2)
Married	35 (27.1)	27 (49.1)	62 (33.7)
Separated	2 (1.6)	2 (3.6)	4 (2.2)
Single	47 (36.4)	10 (18.2)	57 (31)
Widowed	1 (0.8)	0	1 (0.5)

Nationality			
Aboriginal	0	0	0
Torres strait islander	1 (0.8)	0	1 (0.5)
Others	128 (99.2)	55 (100)	183 (99.5)

Previous psychological diagnoses			
Yes	73 (56.6)	24 (43.6)	97 (52.7)
No	56 (43.4)	31 (56.4)	87 (47.3)

Pet owner			
Yes	111 (86)	34 (61.8)	145 (78.8)
No	18 (14)	21 (38.2)	39 (21.2)

Previous horse experience			
Yes	110 (85.3)	42 (76.4)	152 (82.6)
No	19 (14.7)	13 (23.6)	32 (17.4)

Total *N* = 184. *n* = total number of participants in each group. % = percentage of participants in each group.

**Table 2:** Means and standard deviations for perceptions of equine-assisted psychotherapy.

Category	*N*	Mean (M)	Standard deviation (SD)
Health professionals	55	82.62	16.86
Community members	144	96.85	12.13

Dependant variable: perceptions of equine-assisted psychotherapy.

**Table 3:** Predictor correlations of community members perceptions of EAP.

Correlations							
		Social support	Openness	BIRS	Previous horse experience	Pet owner	Location
Social support	Pearson correlation	1	−0.008	0.153	−0.062	0.029	0.103
	Sig. (2-tailed)		0.931	0.082	0.486	0.741	0.245
	*N*	129	129	129	129	129	129

Openness	Pearson correlation	−0.008	1	0.176^*∗*^	0.144	−0.084	0.199^*∗*^
	Sig. (2-tailed)	0.931		0.046	0.104	0.342	0.024
	*N*	129	129	129	129	129	129

BIRS	Pearson correlation	0.153	0.176^*∗*^	1	0.157	−0.139	−0.134
	Sig. (2-tailed)	0.082	0.046		0.076	0.115	0.129
	*N*	129	129	129	129	129	129

Previous horse experience	Pearson correlation	−0.062	0.144	0.157	1	−0.315^*∗∗*^	−0.247^*∗∗*^
	Sig. (2-tailed)	0.486	0.104	0.076		0.000	0.005
	*N*	129	129	129	129	129	129

Pet owner	Pearson correlation	0.029	−0.084	−0.139	−0.315^*∗∗*^	1	0.225^*∗*^
	Sig. (2-tailed)	0.741	0.342	0.115	0.000		0.010
	*N*	129	129	129	129	129	129

Location	Pearson correlation	0.103	0.199^*∗*^	−0.134	−0.247^*∗∗*^	0.225^*∗*^	1
	Sig. (2-tailed)	0.245	0.024	0.129	0.005	0.010	
	*N*	129	129	129	129	129	129

^*∗*^Correlation is significant at the 0.05 level (2-tailed). ^*∗∗*^Correlation is significant at the 0.01 level (2-tailed).

**Table 4:** Hierarchal multiple regression analyses predictors for community members perceptions of EAP.

Unstandardised coefficients			Standardised coefficients	95% CI		
Predictor	B	SE	*β*	LL	UL	sr^2^
Step 1						
Control variables (demographics)						
Diagnosis	2.79	2.23	0.12	−1.63	7.21	0.01
Gender	1.79	2.56	0.07	−3.28	6.86	0.00
Age	−0.08	0.09	−0.10	−0.25	0.09	0.01
Marital status	−1.25	0.87	−0.15	−2.96	0.47	0.02
Education	−1.17	0.70	−0.16	2.55	0.22	0.02
SES	−0.20	0.79	−0.03	−1.77	1.36	0.0

Step 2						
Demographics						
Social support	0.22	0.12	0.16	−0.02	0.45	0.02

Step 3						
Demographics						
Social support	0.21	0.12	0.16	0.02	0.44	0.02
Openness	0.22	0.11	0.18^*∗*^	0.01	0.44	0.03

Step 4						
Demographics						
Social support	0.23	0.12	0.18	−0.01	0.46	0.03
Openness	0.21	0.11	0.17	−0.01	0.42	0.03
Pet ownership	−4.70	2.97	−0.14	−10.57	1.17	0.02

Step 5						
Demographics						
Social support	0.26	0.12	0.20^*∗*^	0.03	0.49	0.03
Openness	0.18	0.11	0.14	−0.04	0.39	0.02
Pet ownership	−2.82	3.06	−0.08	−8.89	3.24	0.01
Previous horse experience	1.21	0.58	0.20^*∗*^	0.05	2.36	0.03

Step 6						
Demographics						
Social support	0.29	0.12	0.23^*∗*^	0.06	0.52	0.04
Openness	0.22	0.11	0.18^*∗*^	0.01	0.44	0.03
Pet ownership	−1.86	3.09	−0.06	−7.97	4.26	0.00
Previous horse experience	0.97	0.59	0.16	−0.20	2.14	0.02
Location	−4.51	2.59	−0.17	−9.64	0.63	0.02

*N* = 129. CI, confidence interval; LL, lower limit; UL, upper limit. 1Sig. values ^*∗*^*P* < 0.05. ^*∗∗*^*P* < 0.01. ^*∗∗∗*^*P* < 0.001. ^a^Dependent variable: community members' perceptions of equine-assisted psychotherapy. ^b^Demographics are collapsed after step one with only predictors of interest shown.

**Table 5:** Predictor correlations of health professionals perceptions of EAP.

		Openness	Previous horse experience	BIRS	Location
Openness	Pearson correlation	1	0.027	−0.011	0.033
	Sig. (2-tailed)		0.848	0.936	0.812
	*N*	55	55	55	55

Previous horse experience	Pearson correlation	0.027	1	0.054	−0.052
	Sig. (2-tailed)	0.848		0.696	0.704
	*N*	55	55	55	55

BIRS	Pearson correlation	−0.011	0.054	1	−0.290^*∗*^
	Sig. (2-tailed)	0.936	0.696		0.032
	*N*	55	55	55	55

Location	Pearson correlation	0.033	−0.052	−0.290^∗^	1
	Sig. (2-tailed)	0.812	0.704	0.032	
	*N*	55	55	55	55

^*∗*^Correlation is significant at the 0.05 level (2-tailed).

**Table 6:** Hierarchal multiple regression analyses predictors for health professionals' perceptions of EAP.

Unstandardised coefficients		Standardised coefficients	95% CI		
Predictor	B	SE	*β*	LL	UL	sr^2^
Step 1						
Control variables						
Age	0.06	0.18	0.05	−0.29	0.42	0.00
Gender	−1.96	4.86	−0.06	−11.72	7.79	0.00

Step 2						
Age	0.04	0.20	0.03	−0.36	0.44	0.00
Gender	−1.93	4.91	0.05	−11.78	7.93	0.00
Openness	1.45	6.10	0.04	−10.80	13.70	0.00

Step 3						
Age	0.04	0.20	0.03	−0.37	0.45	0.00
Gender	−2.05	5.03	−0.06	−12.15	8.06	0.00
Openness	1.50	6.17	0.04	−10.89	13.89	0.00
Previous horse experience	−0.04	0.31	−0.02	−0.66	0.57	0.00

Step 4						
Age	0.08	0.20	0.06	−0.31	0.48	0.00
Gender	−3.08	4.87	−0.09	−12.87	6.71	0.01
Openness	0.26	5.97	0.01	−11.74	12.27	0.00
Previous horse experience	−0.03	0.30	−0.01	−0.62	0.57	0.00
Location	−11.34	5.15	−0.30^*∗*^	−21.70	−0.99	0.09

*N* = 55. CI, confidence interval; LL, lower limit; UL, upper limit. 1Sig. values . ^*∗∗*^*P* < 0.01. ^*∗∗∗*^*P* < 0.001. ^a^Dependent variable: health professionals' perceptions of equine-assisted psychotherapy.

**Table 7:** Means and standard deviations for health Professional's perceptions of equine-assisted psychotherapy (location).

Category	*N*	Mean (M)	Standard deviation (SD)
Rural	1	90.53	12.57
Metropolitan	40	79.65	17.42

Dependent variable: health professional's perceptions of equine-assisted psychotherapy.

## Data Availability

The data used to support the findings of this study are available at https://cloudstor.aarnet.edu.au/plus/s/8OJsk2jk54U1KQy.
